# Measuring milk fat content by random laser emission

**DOI:** 10.1038/srep35119

**Published:** 2016-10-12

**Authors:** Luis M. G. Abegão, Alessandra A. C. Pagani, Sérgio C. Zílio, Márcio A. R. C. Alencar, José J. Rodrigues

**Affiliations:** 1 Departamento de Física, Universidade Federal de Sergipe, 49100-000 São Cristovão, SE, Brazil; 2 Departamento de Tecnologia de Alimentos, Universidade Federal de Sergipe, 49100-000 São Cristovão, SE, Brazil; 3 Instituto de Física de São Carlos, Universidade de São Paulo, CP 369, 13560-970 São Carlos, SP, Brazil; 4 Instituto de Física, Universidade Federal de Uberlândia, Av. João Naves de Ávila 2121, 38400-902 Uberlândia, MG, Brazil

## Abstract

The luminescence spectra of milk containing rhodamine 6G are shown to exhibit typical signatures of random lasing when excited with 532 nm laser pulses. Experiments carried out on whole and skim forms of two commercial brands of UHT milk, with fat volume concentrations ranging from 0 to 4%, presented lasing threshold values dependent on the fat concentration, suggesting that a random laser technique can be developed to monitor such important parameter.

The Random Laser (RL) phenomenon^1^ occurs when a medium emits light with some conventional laser characteristics, such as threshold behavior and spectral narrowing, without being in an optical cavity. In this particular case, light scattering plays a key role, providing incoherent or coherent feedback mechanisms that characterize this emission^2^. Since amplification owing to light diffusion was theoretically considered in the late 60’s^3^, the RL phenomena have been widely studied for potential applications in different fields of knowledge^2^,^4^,^5^. Indeed, random lasers systems are among the most promising light scattering-based tools aiming the development of photonics applications^6^. Here, experiments carried out on whole and skim forms of two commercial brands of UHT milk presented lasing threshold values dependent on the fat concentration, suggesting that a random laser technique can be developed to monitor such important parameter.

Most of the investigated disordered systems that exhibit RL are manmade materials. Controlling the production processes of these synthetic media, the features of the RL could be optimized and several photonic applications proposed. For instance, it is possible to obtain speckle-free images with a low spatial coherence random laser, which was composed by a colloid of polystyrene spheres (240 nm diameter) and laser dye Rhodamine 640 (5 mmol), which was dissolved in diethylene glycol^7^. On the other hand, natural light scattering media, such as human tissue, may also display RL in addition of an amplifying medium. Using the high coherent emission of a random laser it is possible to distinguish malignant human tissue from healthy one^8^. In these two examples, coherent and incoherent feedback RL could be observed and exploited for specific photonic applications. In the latter, the intrinsic disorder of a natural light scattering media was the operation principle of the developed method. These examples demonstrate that RL is a useful tool for the development of characterization techniques and devices based on intrinsic light scattering of natural and artificial systems.

Amongst the myriad of disordered natural media, milk draws attention for being a good example of scattering media^9^ and also because of its importance as nutrient for mammals. While it looks like a uniform turbid liquid in the macroscopic scale, large particles consisting of fat globules, as well as smaller casein micelles appear floating in the plasma. The accurate measurement of these two main components of milk is of great importance for the dairy industry because they determine the price, the food value and compliance with regulatory standards. The typical size of fat globules ranges from 0.1 to 10 micrometers and casein micelles have around 20 to 300 nanometers^10^. This indicates that visible light can scatter according to the Mie process when it propagates in the bulk of such sample and this could lead to RL emission if a gain medium such as rhodamine is present. Spectroscopic experiments and methods involving light scattering^11^ and coherent back scattering^12^ were already studied to determine the properties of milk and its denaturation, respectively. The present work proposes a different spectroscopic method where milk protein and fat globules can be detected using RL.

## Experiments

Most commercial milk appears in two forms, whole and skim, with fat volume concentrations of approximately 3% or more, and 0%, respectively. We measured milks of two UHT commercial brands but since the spectral results were nearly the same, just those of one brand are presented. The composition of milk was determined with two techniques: the Gerger method^13^ and dynamic light scattering (DLS)^14^. The results of the Gerger method showed that the presence of lipids (fat globules) in whole milk were 4.00 and 3.70 grams per each 100 g for each of the two brands. For skim milk, no fat globules were detected with this procedure in each brand. DLS results in whole milk revealed two gaussian size distributions of particles. The average diameter of particles corresponding to the higher intensity distribution (73%) was around 227 nm and for the lower intensity distribution (27%) was 937 nm with a standard deviation of 226 nm. The skim milk presented only one gaussian size distribution with particles of diameters around 220 nm with a standard deviation of 54 nm. These results confirm the presence of fat globules in the whole milk samples, as well its diameter (approximately 930 nm) and its percentage per 100 g (approximately 4%), which are in good agreement with typical features of milk reported previously^10^.

**Figure 1:**
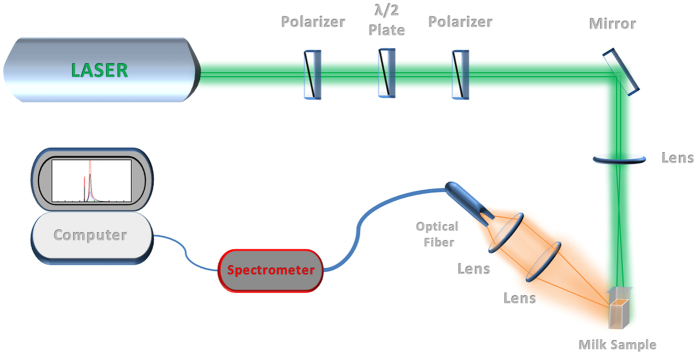
Schematic view of the spectroscopic random laser setup. (Figure made by author L.M.G.A).

Rhodamine 6G (R6G) dye was purchased in analytical solution from Sigma-Aldrich and used with a concentration of 0.1 mM in all solutions. The spectroscopic measurements were performed in fused quartz cuvettes with 10 mm long optical path. The experimental setup is shown in Fig. 1.

The luminescence in our samples was produced with the second harmonic (532 nm) of a Q-switched Nd:YAG laser, delivering 8 ns pulses with a 10 Hz repetition rate. The pulse irradiance was controlled by passing the optical beam through a half-wavelength plate located between two polarizers. The optical beam waist inside the cuvette was estimated to be around 3 mm and the incidence angle was approximately 45 degrees. The light emitted was collected normally to the cuvette surface by a set of lenses coupled to an optical fiber connected to a CCD-compact spectrometer.

## Results and Discussion

**Figure 2:**
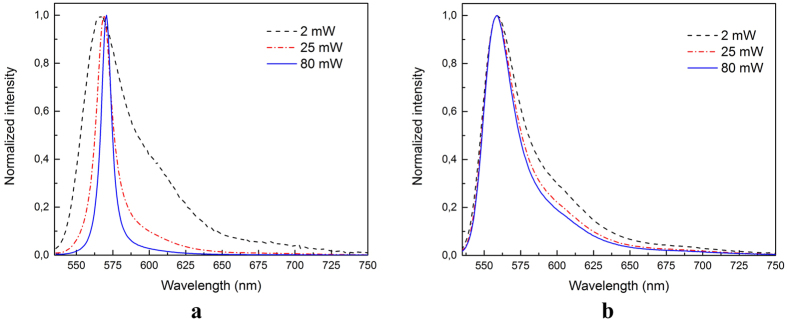
(**a)** Normalized emission spectra of Rhodamine 6G in whole milk brand A and (**b**) in water at excitation average powers of 2 mW (dashed line), 25 mW (dashed-dotted line) and 80 mW (solid line).

At low average powers, the emission spectra of R6G in water and milk are nearly identical, as shown in Fig. 2, with a broadband having a peak located at 564 nm at this concentration^15^. However, the bands in the two brands of whole milk containing R6G get narrower as the average pumping power increases. The luminescence was analyzed by deconvolution of the broadband with two Gaussians. The RL regime observed is associated to the contribution of the monomer emitter^16^. For pumping powers above about 7 mW, the maxima of the milk spectra slightly shifts to longer wavelengths. Furthermore, besides the bandwidth decrease, an increase in the peak intensity is also present.

**Figure 3:**
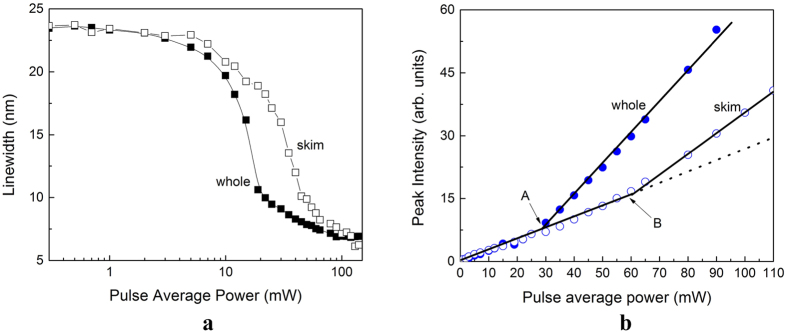
(**a**) Linewidth of whole (solid squares) and skim milk (open squares) in a log scale. (**b**) Emission peak intensity for whole (solid circles) and skim milk (open circles) in a linear scale. The solid lines are just a guide to the eyes.

The results, shown in Fig. 3, indicate that the RL behavior starts first in whole milk, as expected, because of its larger number of scattering centers owing to the fat presence. This behavior is typical of random laser action and must depend on the number of scattering centers present in the emulsion.

In order to confirm this dependence of random laser properties by changing the scattering centers density, we diluted five different ratios of whole milk and skim milk. These dilutions allow us to have five distinct fat concentrations samples, with a constant concentration of 0.1 mM of R6G dye. The diluted ratios were selected to achieve 3.70, 2.78, 1.85, 0.93 and 0.00 grams of fat globules per 100 grams of milk. Because the content of proteins is nearly the same in both whole and skim milks (about 3% in volume), it contributes with the same amount of scattering in all samples. The results revealed that minor scattering center density results in different linewidth and peak intensity thresholds, as expected from the RL theory and previous studies^17^.

**Figure 4:**
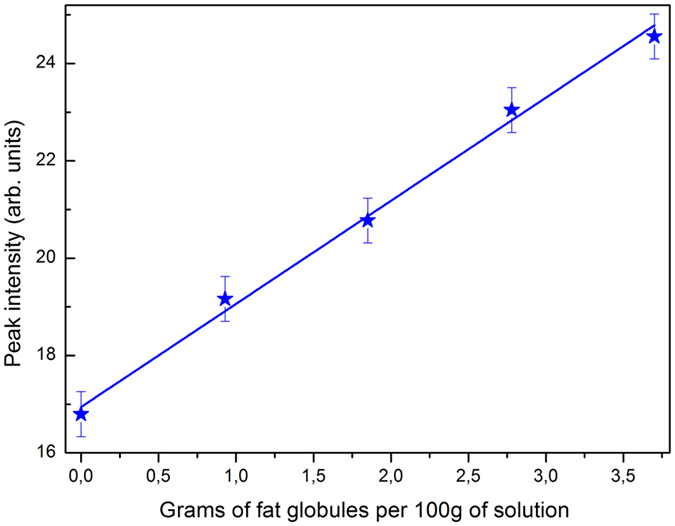
Emission peak intensity at five different fat globules concentrations.

Hence, in order to develop a practical instrument to determine the fat content we analyzed the peak height of the band centered around 564 nm of those five samples, employing a power of 60 mW, shown in Fig. 4, which is approximately above the intensity threshold of all samples. These results demonstrate that the measurement of the RL emission peak intensity would provide a relatively cheap and practical approach for the development of a sensor to determine fat concentration in milk. Although we used here a spectrometer, a bandpass filter centered around 564 nm could alternatively be employed to determine how the RL emission is sensible to the milk fat concentration.

We observed RL behavior in UHT milk and since the results presented lasing threshold values dependent on the fat concentration, a technique to monitor such parameter could be developed. Further experiments still need to be done to understand how this method applies to non-homogenized natural milk.

## Method

Gerber method is based on milk emulsion breaking by the addition of sulfuric acid (Specific gravity 1.820–1.825) and isoamyl alcohol (Specific gravity 0.814–0.816). The sulfuric acid digests proteins and carbohydrates. The isoamyl alcohol generally prevents the charring of sugar.

The procedure steps to determine the fat concentration were: (1) Transfer 10 mL of H_2_SO_4_ (Specific gravity 1.820–1.825) at 15–21 °C into a Gerber milk butyrometers (graduated 0–8%); (2) Accurately measure milk sample (11 mL) into the Gerber Butyrometers, using a Gerber pipette; (3) Add 1 mL isoamyl alcohol (Specific gravity 0.814–0.816) to the Butyrometers; (4) Tighten the stopper and mix by shaking the butyrometer; (5) Centrifuge the butyrometer at (1150 ± 70) rpm for 5 min; (6) Place the butyrometer in a water bath at (63 ± 2) °C for 3 minutes; (7) Finalizing was read percentage of fats directly on the graduated scale of the butyrometer. All analyzes were performed in triplicate.

## Additional Information

**How to cite this article**: Abegão, L. M. G. *et al*. Measuring milk fat content by random laser emission. *Sci. Rep.* **6**, 35119; doi: 10.1038/srep35119 (2016).
